# Effects of hypoglycaemia on working memory and regional cerebral blood flow in type 1 diabetes: a randomised, crossover trial

**DOI:** 10.1007/s00125-017-4502-1

**Published:** 2017-11-29

**Authors:** Michael Gejl, Albert Gjedde, Birgitte Brock, Arne Møller, Eelco van Duinkerken, Hanne L. Haahr, Charlotte T. Hansen, Pei-Ling Chu, Kirstine L. Stender-Petersen, Jørgen Rungby

**Affiliations:** 1 Department of Biomedicine, Aarhus University, Bartholins Allé 6, Building 1242, 8000 Aarhus C, Denmark; 2 Department of Endocrinology and Internal Medicine, Aarhus University Hospital, Aarhus, Denmark; 3 Department of Neuroscience, University of Copenhagen, Copenhagen, Denmark; 4 Department of Clinical Medicine, University of Southern Denmark, Odense, Denmark; 5 Department of Clinical Biochemistry, Aarhus University Hospital, Aarhus, Denmark; 6 Steno Diabetes Center Copenhagen, Gentofte, Denmark; 7 PET-Center, Department of Nuclear Medicine, Aarhus University Hospital, Aarhus, Denmark; 8 VU University Medical Centre, Amsterdam, the Netherlands; 9 Pontifícia Universidade Católica, Rio de Janeiro, Brazil; 10 Novo Nordisk A/S, Søborg, Denmark; 11 Novo Nordisk Inc., Plainsboro, NJ USA; 12 Department of Endocrinology IC, Bispebjerg University Hospital, Bispebjerg, Copenhagen, Denmark

**Keywords:** Clinical diabetes, Hypoglycaemia, Imaging (MRI/PET/other), Insulin therapy

## Abstract

**Aims/hypothesis:**

The aim of this randomised, crossover trial was to compare cognitive functioning and associated brain activation patterns during hypoglycaemia (plasma glucose [PG] just below 3.1 mmol/l) and euglycaemia in individuals with type 1 diabetes mellitus.

**Methods:**

In this patient-blinded, crossover study, 26 participants with type 1 diabetes mellitus attended two randomised experimental visits: one hypoglycaemic clamp (PG 2.8 ± 0.2 mmol/l, approximate duration 55 min) and one euglycaemic clamp (PG 5.5 mmol/l ± 10%). PG levels were maintained by hyperinsulinaemic glucose clamping. Cognitive functioning was assessed during hypoglycaemia and euglycaemia conditions using a modified version of the digit symbol substitution test (mDSST) and control DSST (cDSST). Simultaneously, regional cerebral blood flow (rCBF) was measured in pre-specified brain regions by six H_2_^15^O-positron emission tomographies (PET) per session.

**Results:**

Working memory was impaired during hypoglycaemia as indicated by a statistically significantly lower mDSST score (estimated treatment difference [ETD] −0.63 [95% CI −1.13, −0.14], *p* = 0.014) and a statistically significantly longer response time (ETD 2.86 s [7%] [95% CI 0.67, 5.05], *p* = 0.013) compared with euglycaemia. During hypoglycaemia, mDSST task performance was associated with increased activity in the frontal lobe regions, superior parietal lobe and thalamus, and decreased activity in the temporal lobe regions (*p* < 0.05). Working memory activation (mDSST − cDSST) statistically significantly increased blood flow in the striatum during hypoglycaemia (ETD 0.0374% [95% CI 0.0157, 0.0590], *p* = 0.002).

**Conclusions/interpretation:**

During hypoglycaemia (mean PG 2.9 mmol/l), working memory performance was impaired. Altered performance was associated with significantly increased blood flow in the striatum, a part of the basal ganglia implicated in regulating motor functions, memory, language and emotion.

**Trial registration:**

NCT01789593, clinicaltrials.gov

**Funding:**

This study was funded by Novo Nordisk.

**Electronic supplementary material:**

The online version of this article (10.1007/s00125-017-4502-1) contains peer-reviewed but unedited supplementary material, which is available to authorised users.

## Introduction

Cognitive performance, even during simple tasks, is impaired during acute hypoglycaemia (plasma glucose [PG] <2.5 mmol/l [45.0 mg/dl]) in individuals with type 1 diabetes mellitus [1, 2] and the degree of impairment depends on the level of hypoglycaemia [3]. Additionally, the cognitive impairment induced by hypoglycaemia (PG 2.5–2.7 mmol/l) can remain following the restoration of euglycaemia [4–6]; with some studies showing cognitive impairments lasting for approximately 45–75 min after euglycaemic restoration [7, 8]. Working memory is an important aspect of cognitive function and is susceptible to the effects of hypoglycaemia. It has been demonstrated using functional MRI (fMRI) that, compared with healthy individuals, people with type 1 diabetes mellitus require higher levels of brain activation to attain parity for working memory performance during hypoglycaemia (PG ≤2.8 mmol/l [50.4 mg/dl]) [9]. Thus, identification of cerebral activation patterns during working memory performance at different PG levels could enhance our understanding of mechanisms underlying the reduced cerebral efficiency seen in type 1 diabetes mellitus [9]. For example, it remains to be clarified if milder hypoglycaemic episodes, PG just below 3.1 mmol/l and previously associated with altered brain activity [10, 11], have a similar negative impact on working memory to those demonstrated at PG ≤2.8 mmol/l. In individuals with type 1 diabetes mellitus, cerebral blood flow (CBF) and cerebral glucose metabolism [12] within total grey matter are correlated. This correlation becomes stronger when adjusted for glucose levels, allowing CBF assessment to be used as a proxy for cerebral metabolism [13]. However, there are some limitations to assessment with fMRI; namely it does not provide a direct measure of neuronal oxygen consumption or neuronal activation and results are vulnerable to movement distortion. Conversely, radiolabelled water (H_2_^15^O) positron emission tomography (PET) is a direct measure of cerebral oxygen consumption (and thus neuronal activation) that is less affected by movement and more quantifiable than fMRI [14]. This hypothesis-driven study aimed to test if cognitive performance (assessed by H_2_^15^O PET) and associated CBF estimates are affected at less pronounced levels of hypoglycaemia than previously studied and if cognitive performance is affected in the recovery phase following less pronounced hypoglycaemia.

## Methods

### Study design

This randomised, single-centre, patient-blinded, two-period crossover study compared cognitive performance (assessed by working memory performance and reaction time) under hypoglycaemic (aiming for a PG target just below 3.1 mmol/l; the clamp target was defined as 2.8 ± 0.2 [2.6–3.0] mmol/l) and euglycaemic (PG clamp target 5.5 mmol/l ± 10%) conditions in participants with type 1 diabetes mellitus (ESM Fig. 1). Blood was drawn at pre-specified time points to assess counter-regulatory hormone responses. Hypoglycaemia awareness and symptoms during both clamps were also assessed. Participants underwent the sequence of glycaemic conditions in a blind and randomised order determined by sequential enrolment and lowest available number assignment. The two experimental visits were separated by 21–42 days (to avoid effects of counter-regulatory hormone responses or other physiological effects of hypoglycaemia). Female participants attended the two visits at the same stage of their menstrual cycle. The study was conducted from 14 January to 1 December 2013 at the Department of Endocrinology and Department of Nuclear Medicine and PET Center, Aarhus University Hospital, Aarhus, Denmark. Informed written consent was obtained from all participants before any study-related activities. The study was conducted in compliance with International Conference on Harmonisation Good Clinical Practice [15], the Declaration of Helsinki [16] and was approved according to local regulations by an independent ethics committee.

### Participants

Inclusion criteria for participants screened (*n* = 37) were right-handedness, age 18–64 years, BMI 18.0–28.0 kg/m^2^, HbA_1c_ ≤9.0% (≤75 mmol/mol), diagnosed with type 1 diabetes mellitus and treated with multiple daily insulin injections or continuous subcutaneous insulin infusion for ≥12 months prior to screening. Key exclusion criteria included known central nervous system abnormalities, structural brain abnormalities (identified by structural MRI scans during screening), severe hypoglycaemia (requiring third party assistance) or ketoacidosis in the last 6 months, clinically defined hypoglycaemic unawareness, and treatment with medications potentially interfering with glucose metabolism. Key experimental visit exclusion criteria included occurrence of a hypoglycaemic event (with PG ≤3.9 mmol/l) within the preceding 48 h. Full inclusion and exclusion criteria are listed in ESM Table 1.

### Cognitive tests

The Wechsler Adult Intelligence Scale (third edition) revised digit symbol substitution test (DSST) [17] was previously adapted and validated to specifically measure working memory [18], and has been successfully used with fMRI [19]. Here, we used the stimuli of this adapted DSST paradigm and modified it to our specific PET procedure. As with the fMRI design, we used the modified DSST (mDSST) task to measure working memory. A control task (cDSST) was used for non-task-related brain activation (without a working memory load), including visual and motor cortex activation (for eye sight and movement) and index finger movement.

Subtraction of regional CBF (rCBF) patterns during cDSST from those during mDSST was interpreted as indicative of brain activation patterns exclusively associated with the operation of working memory. The mDSST task consisted of three blocks of 32 randomly presented digit–symbol combinations (ESM Methods: Cognitive tests – mDSST and cDSST task combination blocks and ESM Fig. 2), fitting the specific H_2_^15^O PET design. The cDSST task had the same basic design. Each block began with test instructions shown for 7.8 s and was 0.2 s longer than every 3 min PET acquisition, to ensure participants were engaged in the mDSST or cDSST task during the full 3 min PET acquisition. Tasks were presented using E-prime 2.0 (Psychology Software Tool, Pittsburgh, PA, USA) through audiovisual goggles inside the PET scanner. Correct responses (no response was considered as incorrect) and response time were recorded and analysed.

To test working memory following a prolonged recovery phase (75–90 min after PG of 5–6 mmol/l was restored) following hypoglycaemia or euglycaemia, the paced auditory serial addition task (PASAT) was preferred over the mDSST to avoid bias from habituation [20, 21]. During the PASAT, participants heard a digit and had to add the next digit (presented 3 or 2 s later) and report the sum aloud. Both parts consisted of 60 digits and correct responses were recorded.

### PET imaging

Each participant underwent PET imaging at both visits. First, a 6 min transmission scan for attenuation correction was performed. Thereafter, six 3 min tomography sessions with either mDSST or cDSST in a fixed order were performed. Cerebral activity levels were measured as change in brain uptake of radiolabelled water (H_2_^15^O), the retention of which matches the rate of CBF, by means of a high-resolution research tomograph (Siemens/CTI, Knoxville, TN, USA) operating in 3-D mode. Additional detail is provided in ESM Methods: PET imaging.

### Experimental visit procedures

**Fig. 1:**
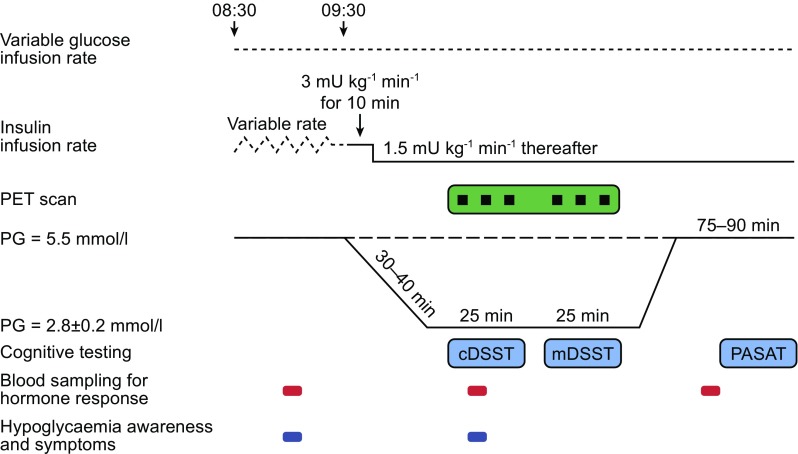
Experimental visit design

Participants attended the study site at approximately 20:00 hours on the day before each experimental procedure, at which point normal insulin treatment was suspended. Participants stayed overnight to ensure stabilisation of PG within the range of 5–8 mmol/l via variable intravenous infusion of insulin (Actrapid®, 100 U/ml) and glucose (20% glucose/dextrose/10 mmol/l KCl), before initiation of experimental procedures at 08:00 hours the following day. On the days of the experimental procedures, cognitive tests were briefly performed ≥1 h before initiation of hypoglycaemia or euglycaemia to prevent practice effects. Each glycaemic condition was preceded by a 60 min run-in period whereby variable intravenous infusions of glucose or human soluble insulin were delivered to obtain a steady-state PG target level of 5.5 mmol/l ± 10%. During the run-in and clamps, the participants’ cannulated hand was placed in a thermoregulated box with their arterialised venous blood sampled for PG measurements using a benchtop glucose analyser (YSI 2300 Stat Plus, Yellow Springs, OH, USA). Euglycaemia was maintained using a glucose clamp (glucose/Actrapid® infusion) for approximately 1 h after which insulin (Actrapid®, 100 U/ml) was given at an infusion rate of 3 mU kg^−1^ min^−1^ for 10 min and then reduced to 1.5 mU kg^−1^ min^−1^ thereafter. Euglycaemia was maintained or hypoglycaemia induced with glucose infusion rate adjusted accordingly to meet the PG target for approximately 55 min, during which H_2_^15^O PET scans and cDSST and mDSST tasks were performed (Fig. 1). Following glycaemic clamps, participants were brought back to euglycaemia (with glucose infusions to reach a PG target of 5–6 mmol/l) and after approximately 75–90 min the PASAT was conducted. After experimental procedures, participants resumed usual insulin treatment.

### Counter-regulatory hormones

Counter-regulatory hormones were measured as a validation that the PG target was sufficient to elicit a counter-regulatory response, and thus, hypoglycaemia. Hormonal responses (noradrenaline [norepinephrine], glucagon, cortisol and growth hormones) were measured 30 min prior to induction of hypoglycaemia or euglycaemia, and 45 min and 150 min (just prior to PASAT test) after induction of hypoglycaemia or euglycaemia.

### Hypoglycaemia awareness and symptoms

At screening, hypoglycaemia unawareness was assessed by asking participants ‘Can you feel your hypos?’ and checking their medical files for any indication of unawareness. During the study, hypoglycaemia awareness was assessed both 30 min prior to and 45 min after induction of hypoglycaemia or euglycaemia, by asking participants ‘Do you feel hypo?’. The hypoglycaemia symptoms questionnaire (based on the Edinburgh Condition Scale) [22] was completed by participants 30 min prior to and 55 min after induction of hypoglycaemia or euglycaemia. It measured autonomic (sweating, palpitations, shaking and hunger), neuroglycopenic (confusion, drowsiness, odd behaviour, speech difficulty and incoordination) and general malaise (headache and nausea) symptoms on a seven point Likert scale.

### rCBF assessment

Cerebral activation was measured as rCBF in 19 pre-specified regions of interest (ROI). Given the hypothesis-driven nature of this study, these regions were selected in accordance with relevant literature for one or more of the following criteria: DSST evoked brain activity patterns in normal conditions, i.e. euglycaemia, without working memory load (precuneus, dorsolateral prefrontal cortex, anterior cingulate gyrus/cortex, posterior cingulate gyrus, posterior supramarginal gyrus and orbitofrontal cortex) [19, 23]; neural substrate of DSST performance (inferior frontal gyrus, superior frontal gyrus, middle frontal gyrus, superior parietal lobe, precuneus, posterior cingulate gyrus/cortex and parahippocampal gyrus) [24, 25]; brain areas showing changes in functional activity in response to hypoglycaemia (medial temporal lobe, hippocampus, parahippocampal gyrus, insula, globus pallidum, striatum, dorsolateral prefrontal cortex, anterior cingulate gyrus, inferior frontal gyrus, superior frontal gyrus, precuneus, posterior cingulate gyrus, posterior supramarginal gyrus and primary visual cortex) [9, 26–30]; and brain areas reported as involved in working memory tasks (dorsolateral prefrontal cortex, inferior frontal gyrus, middle frontal gyrus, superior frontal gyrus, ventromedial prefrontal cortex, orbitofrontal cortex, insula, superior parietal lobe, anterior cingulate gyrus/cortex, hippocampus and thalamus) [31–33]. All rCBF measures were normalised to measures in the cerebral cortex, as this region is considered to be less impacted by the duration of type 1 diabetes mellitus [34]. In the present study, there was no significant difference in rCBF in the cerebral cortex between hypoglycaemia and euglycaemia during either cDSST or mDSST tasks.

Three endpoints were used to determine regional cerebral activation: rCBF during mDSST and cDSST performances, and rCBF for working memory. For the cDSST and mDSST endpoints, rCBF was calculated by subtracting the mean of three rCBF values for euglycaemia from the mean of three rCBF values for hypoglycaemia. To isolate changes as a result of working memory, rCBF values during the mDSST test were corrected for the rCBF values during cDSST, by subtracting the mean of three cDSST rCBF values from the mean of three mDSST rCBF values; this correction was conducted for measurements taken during both glycaemic clamps with the totals for euglycaemia subtracted from those for hypoglycaemia ([mean (3 × CBF during mDSST) − mean (3 × CBF during cDSST)] hypoglycaemia − [mean (3 × CBF during mDSST) − mean (3 × CBF during cDSST)] euglycaemia) to isolate changes in working memory during hypoglycaemia.

### Endpoints and statistical analyses

The primary objective of the study was to compare cognitive performance during hypoglycaemia with that during euglycaemia. The primary endpoint was the number of correct mDSST scores. For each glycaemic condition, mean mDSST scores, reaction time and PASAT scores were compared using a linear mixed-effect model with glycaemic condition and period as fixed factors and participant as a random factor; mean differences between hypoglycaemia and euglycaemia were estimated from the model and corresponding 95% CI and *p* values were calculated. The rCBF and predefined ROI (during both cDSST and mDSST), as well as the difference in rCBF between the two tasks, were compared during glycaemic conditions using an analysis of variance with glycaemic condition, period and participant as fixed factors. Because of the hypothesis-driven nature of this trial, no correction for multiplicity was performed with regard to different ROI analyses.

The SD for DSST score between euglycaemia and hypoglycaemia (PG 2.5 mmol/l) was determined in a previous trial (NCT01002768) to be approximately nine. Assuming a similar variability in this trial, using a 5% significance level and two-sided paired *t* test, a sample size of 25 participants completing both periods was calculated to have 90% power to detect a true difference in DSST score between hypoglycaemia and euglycaemia of approximately six. A total of 28 participants were randomised to ensure at least 25 participants completing both experimental visits.

## Results

### Participant disposition

Of the 37 participants screened, 29 (22 men and seven women) were randomised and 26 completed both experimental visits (ESM Fig. 3); data are presented for completers only (Table 1). Three participants were withdrawn after randomisation (two men and one woman) for inability to place the venous catheter (*n* = 1), erroneous randomisation (*n* = 1) and meeting experimental visit exclusion criteria (*n* = 1). Mean age of participants was 38.7 years (range 19.0–65.1 years), BMI was 24.6 kg/m^2^, HbA_1c_ was 7.3% (56.7 mmol/mol) and duration of diabetes was 18.7 years (Table 1). The educational level of participants was a mean ± SD of 14.0 ± 2.0 years (Table 1). Individual PG profiles during hypoglycaemic and euglycaemic clamps are shown in ESM Fig. 4. Mean ± SD PG achieved during hypoglycaemia was 2.9 ± 0.14 mmol/l, which is within the target PG. During the study four adverse events (AEs) were reported in three participants (two each during hypoglycaemia [presyncope and orthostatic hypotension] and euglycaemia [headache and nausea]). All AEs were non-serious, mild and classified as not related to Actrapid or devices used. One participant reported an AE after release from the trial site (flank pain).

**Table 1:** Baseline characteristics of completers

Characteristic	Value
Number of participants	26
Age, years (mean ± SD)	38.7 ± 15.3
BMI, kg/m^2^ (mean ± SD)	24.6 ± 2.7
Race	
Of European descent, *n* (%)	26 (100.0)
Sex	
Female, *n* (%)	6 (23.1)
Male, *n* (%)	20 (76.9)
Educational level, years (mean ± SD [min–max])	14.0 ± 2.0 (10.0–18.0)
Diabetes characteristics (mean [min–max])	
Duration of diabetes, years	18.7 (3.1–46.4)
HbA_1c_, mmol/mol	56.7 (39.0–74.0)
HbA_1c_, %	7.3 (5.7–8.9)

Baseline information was recorded at screening and/or randomisation. If an assessment was recorded on both visits, the randomisation value was used as the baseline value

### Cognitive performance

**Fig. 2:**
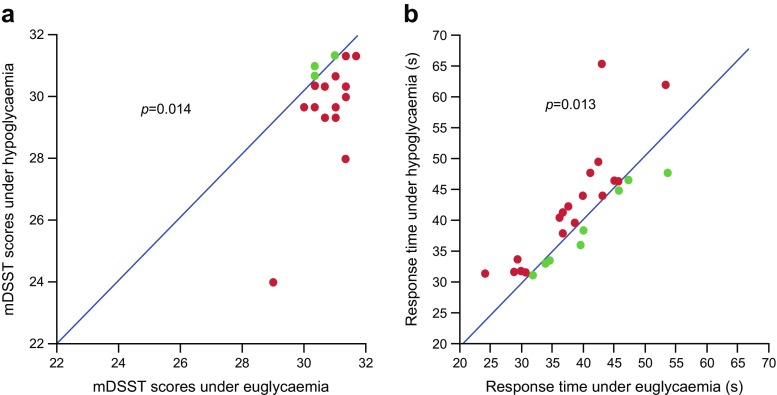
(**a**) Observed mDSST scores and (**b**) response times in hypoglycaemia and euglycaemia. Only participants with available endpoints for both conditions were included in the statistical analysis. The endpoint was analysed using a linear mixed-effect model with condition (hypoglycaemia or euglycaemia) and period as fixed factors, and participant as a random factor. (**a**) Red dots represent participants where the correct number of mDSST responses is higher during euglycaemia than hypoglycaemia. Green dots represent participants where the correct number of mDSST responses was higher during hypoglycaemia than euglycaemia. ETD hypoglycaemia – euglycaemia −0.63 (95% CI −1.13, −0.14). Number of correct responses (observed mean ± SD: hypoglycaemia, 30.3 ± 1.5; euglycaemia, 30.9 ± 0.6). (**b**) Red dots represent participants where the mDSST response time is longer during hypoglycaemia than euglycaemia. Green dots represent participants where the mDSST response time is longer during euglycaemia than hypoglycaemia. ETD hypoglycaemia – euglycaemia 2.86 (95% CI 0.67, 5.05). Total response time (observed mean ± SD: hypoglycaemia, 41.5 ± 8.9 s; euglycaemia, 38.7 ± 7.3 s)

On average, participants had a statistically significantly lower mean mDSST score (± SD) during hypoglycaemia than euglycaemia (30.3 ± 1.5 vs 30.9 ± 0.6), with an estimated treatment difference (ETD) of −0.63 (95% CI −1.13, −0.14; *p* = 0.014) (Fig. 2a). Mean ± SD total response time during mDSST and hypoglycaemia was significantly longer than during euglycaemia (41.5 ± 8.9 s vs 38.7 ± 7.3 s), with an ETD of 2.86 s (95% CI 0.67, 5.05; *p* = 0.013) (Fig. 2b). Exclusion of two outliers (Fig. 2 and ESM Table 2) from the statistical analysis did not affect outcomes, with the mDSST score remaining significantly lower (ETD −0.32 [95% CI −0.62, −0.03], *p* = 0.035) and total response time remaining longer during hypoglycaemia compared with euglycaemia (ETD 2.00 s [95% CI 0.48, 3.52], *p* = 0.012). For working memory in the recovery phase, PASAT scores were not statistically significantly different following hypoglycaemia compared with euglycaemia, for either the 3 s PASAT (ETD −0.37 [95% CI −3.26, 2.52], *p* = 0.794) or the 2 s PASAT (ETD 0.79 [95% CI −1.34, 2.91], *p* = 0.454; ESM Table 3).

### rCBF

#### Effects of hypoglycaemia on rCBF during cDSST

**Fig. 3:**
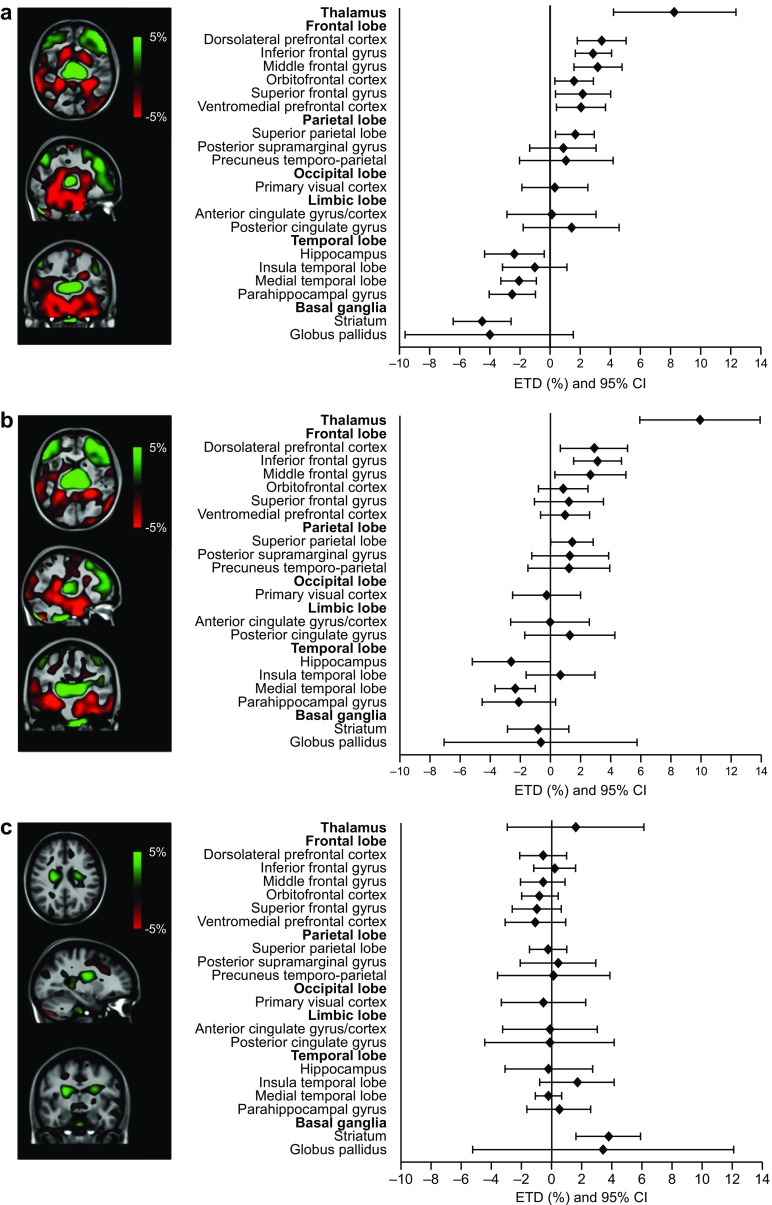
Normalised rCBF from PET scans and associated forest plots showing differences in brain activation in hypoglycaemia compared with euglycaemia during (**a**) cDSST, (**b**) mDSST and (**c**) mDSST − cDSST. Only participants with available endpoints for both conditions were included in the analysis. The PET scans and forest plots are normalised to the cerebral cortex and show the percentage difference in rCBF in hypoglycaemia compared with euglycaemia (green, increase in rCBF; red, decrease in rCBF). Endpoints were compared using an ANOVA model with condition, period and participant as fixed factors

During the cDSST, without a working memory load, rCBF was statistically significantly lower during hypoglycaemia compared with euglycaemia in three temporal lobe regions (hippocampus, medial temporal lobe and parahippocampal gyrus) and in the striatum. In addition, rCBF was statistically significantly increased in six frontal lobe regions (dorsolateral prefrontal cortex, inferior frontal gyrus, middle frontal gyrus, orbitofrontal cortex, superior frontal gyrus and ventromedial prefrontal cortex), the superior parietal lobe and in the thalamus (Fig. 3a and ESM Table 4).

#### Effects of hypoglycaemia on rCBF during mDSST

During the mDSST (with a working memory load) rCBF was statistically significantly increased in three frontal lobe regions (dorsolateral prefrontal cortex, inferior frontal gyrus and middle frontal gyrus), the superior parietal lobe and the thalamus, and statistically significantly decreased in the hippocampus and the medial temporal lobe during hypoglycaemia (Fig. 3b and ESM Table 4).

#### Differences in rCBF between hypoglycaemia and euglycaemia during cDSST and mDSST

Isolating working memory (measured as the rCBF during mDSST corrected for cDSST), there was statistically significantly higher blood flow in the striatum during hypoglycaemia when compared with euglycaemia (Fig. 3c), with an ETD of 0.0374% (95% CI 0.0157%, 0.0590%; *p* = 0.002).

### Hypoglycaemia assessments and counter-regulatory hormone responses

When asked ‘Do you feel hypo?’ 30 min prior to induction of glycaemic conditions, the proportion of participants responding ‘no’ was 96.2% and 92.3%, for those exposed to prior hypoglycaemia or euglycaemia, respectively. During the glycaemic clamps (nominal time 45 min) proportionally more participants answered ‘yes’ when they were asked if they felt hypoglycaemic during hypoglycaemia (42.3%) compared with euglycaemia (11.5%). When we compared these subgroups in a post hoc analysis to determine the impact on the primary endpoint (mDSST score and response times), we found no differences between those responding ‘yes’ or ‘no’ to feeling the experimental hypoglycaemia (ESM Results). Mean ± SD hypoglycaemic symptom scores 30 min prior to induction of hypoglycaemia and euglycaemia were 17.69 ± 5.67 and 18.69 ± 4.88, respectively. Hypoglycaemic symptom scores during hypoglycaemia (55 min after initiating the induction of hypoglycaemia) and euglycaemia were 23.38 ± 11.67 and 18.27 ± 4.75, respectively.

**Fig. 4:**
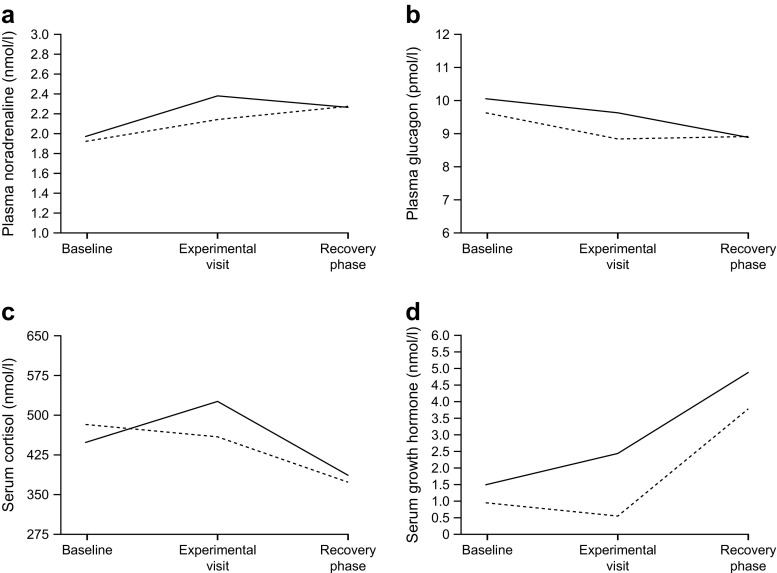
Counter-regulatory hormonal responses for (**a**) noradrenaline, (**b**) glucagon, (**c**) cortisol and (**d**) growth hormone. Analysis was based on completers. Solid line represents hypoglycaemia; dashed line represents euglycaemia

Noradrenaline, cortisol and growth hormone responses were increased during hypoglycaemia compared with euglycaemia. The response of glucagon was compromised when comparing hypoglycaemia with euglycaemia. The counter-regulatory hormonal responses are shown in Fig. 4.

## Discussion

This multidisciplinary study examined how cognitive performance and its concurrent rCBF were affected by hypoglycaemia at a mean PG of 2.9 mmol/l. This is a higher level of PG than has been tested in previous studies [1, 2], but matches the level recommended in reporting of clinical trials by the recent position statement from the International Hypoglycaemia Study Group [35] and is considered to be ‘sufficiently low to indicate serious, clinically important hypoglycaemia’. During hypoglycaemia there was a modest, yet significant, decrease in the number of correct mDSST responses referring to working memory, as well as a significant increase in response time. The effects of hypoglycaemia on rCBF in the striatum was statistically significant, highly uniform and with little variation (in SD) between participants. Furthermore, use of working memory during hypoglycaemia was associated with a significant increase in blood flow in the striatum.

Previous studies have reported reductions in cognitive function scores in the range of 2–40% during hypoglycaemia [8, 36]. The comparatively small decrease in number of correct mDSST responses in our study indicates, as expected, that working memory function was relatively preserved with regard to accuracy but at the cost of response speed [3]. These results indicate that during hypoglycaemia, at a mean PG of 2.9 mmol/l, participants with type 1 diabetes mellitus may be able to keep performance at a similar level as during euglycaemia, at least when performing less complex tasks, but at a 7% slower execution rate (3 s over a block of 32 questions). While this level of slowing may not be of great importance for many daily functions where response time is not critical, it could become serious in tasks which do depend on rapid information processing, such as driving.

Our study showed no difference in the number of correct PASAT responses in the recovery phase following hypoglycaemia compared with euglycaemia, possibly reflecting time of testing (75–90 min after euglycaemia restoration). Cognitive performance during recovery from hypoglycaemia has varied between different studies [4–6]. In one study, the cognitive tests were repeated at 10–15 min intervals up to 85 min after restoration of euglycaemia, and cognitive performance, for some tasks, was found to be only impaired for up to 10 min after euglycaemia was restored [6].

During the hypoglycaemic clamp, approximately 58% of participants answered ‘no’ to feeling hypoglycaemic, despite efforts made to exclude participants with impaired awareness of hypoglycaemia. Despite this, rCBF was significantly impacted during both cDSST and mDSST tasks. Indeed, both were associated with increased rCBF in frontal lobe regions (goal-directed action, behavioural control and problem solving) [34, 37] and the thalamus (relay station) and decreased rCBF in temporal lobe regions (memory functions). Furthermore, some regions that were deactivated during cDSST (and hence received reduced rCBF) were less affected during mDSST when working memory function was required; hence, rCBF was also higher in the basal ganglia and insula.

The effects of hypoglycaemia on rCBF in the thalamus, insula and globus pallidus were not statistically significantly different between cDSST and mDSST, but the effect in the striatum was significantly different and was highly uniform across participants, as indicated by the narrow confidence intervals (Fig. 3c).

In people with type 1 diabetes mellitus, studies have revealed increased CBF in the hypothalamus, brainstem, anterior cingulate cortex and putamen when reducing PG from 5.2 to 4.3 mmol/l. This suggests that these regions are sensitive to small blood glucose changes, and that even small sudden glycaemic changes may be of clinical importance [11]. In the current study, increased rCBF in the frontal lobe areas during hypoglycaemia could reflect the processing of input from the thalamus, which was also highly activated. Working memory was associated with increased blood flow in the striatum, a part of the basal ganglia thought to support motor functions, memory, language and emotion regulation. Increased rCBF in this region may suggest an increased support of this subcortical system during a task that is otherwise related to frontal regions, suggesting that the brain requires more resources to maintain performance during hypoglycaemia. A less efficient brain (i.e. recruiting more resources to preserve cognitive performance) during hypoglycaemia (PG ≤2.8 mmol/l) in individuals with type 1 diabetes mellitus was also shown by a previous study that used a working memory task during fMRI [9].

### Study limitations

Regional blood flow averages did not allow testing for significant changes of blood flow in smaller parts of individual brain regions; this would require specific voxel-based analysis. A further limitation is that individual responses to hypoglycaemia can vary widely [38], as illustrated here by the finding that about half of participants had an impaired awareness of hypoglycaemia during the clamp and thus did not feel the impact of hypoglycaemia during the hypoglycaemic clamp. This may be attributed to the milder levels of hypoglycaemia achieved in the study (PG 2.9 mmol/l) and the comparable threshold for appearance of symptoms [39, 40]; however, it may also reflect the inclusion of participants with previously unrecognised unawareness and an inability to identify reduced awareness in everyday life. A more complete picture of potential impairments in counter-regulatory hormonal responses would have been afforded if adrenaline (epinephrine) had also been measured. The cDSST and mDSST tasks were not alternated and therefore the rCBF response to hypoglycaemia may be subject to time-dependent differences in activation. Although a time-dependent impact of hypoglycaemia on the brain has been suggested [29], H_2_^15^O PET imaging for the cDSST and mDSST tasks commenced after the target hypoglycaemic PG was reached (0–20 min and 30–60 min, respectively). Therefore, effects of decreasing PG on the brain were not captured in this study and will limit any potential time-dependent effect. Finally, no cognitive dysfunction screening was carried out on participants prior to the study; however, cognitive problems related to working memory or other memory functions are not typically seen in adults with type 1 diabetes mellitus [41]. Despite these limitations the data still challenge the prevailing notion that working memory is not impaired until glucose levels are <2.8 mmol/l and shows that working memory is also impaired between 2.8 and 3.0 mmol/l, the glucose levels that generally reflect the lower and upper limits for the manifestation of hypoglycaemia symptoms [39, 40].

In conclusion, this hypothesis-driven study demonstrated that working memory performance and reaction times are adversely affected by hypoglycaemia in individuals with type 1 diabetes mellitus, and that they are associated with measurable effects on rCBF, even at mean PG concentrations of 2.9 mmol/l. The findings are clinically relevant, supporting the importance of reducing the risk of hypoglycaemic episodes at this level.

## Electronic supplementary material

ESM (PDF 271 kb)

## Abbreviations

AE: Adverse event
CBF: Cerebral blood flow
cDSST: Control digit symbol substitution test
DSST: Digit symbol substitution test
ETD: Estimated treatment difference
fMRI: Functional MRI
mDSST: Modified digit symbol substitution test
PASAT: Paced auditory serial addition task
PET: Positron emission tomography
PG: Plasma glucose
rCBF: Regional cerebral blood flow
ROI: Regions of interest
