# Supplementary material for: Effects of hypoglycaemia on working memory and regional cerebral blood flow in type 1 diabetes: a randomised, crossover trial
Source: Diabetologia. 2017 Nov 29;61(3):551–61. doi: 10.1007/s00125-017-4502-1 (PMC6448973; doi:10.1007/s00125-017-4502-1)
Supplement: Supplementary file 1 — (PDF 271 kb) [file 125_2017_4502_MOESM1_ESM.pdf]

Electronic supplementary material

Effects of hypoglycaemia on working memory and regional cerebral blood flow in type 1 diabetes: a randomised, crossover trial

Authors: Michael Gejl, Albert Gjedde, Birgitte Brock, Arne Møller, Eelco van Duinkerken, Hanne L. Haahr, Charlotte T Hansen, Pei-Ling Chu, Kirstine L Stender-Petersen, Jørgen Rungby

ESM methods

Cognitive tests – modified version of the digit symbol substitution test (mDSST) and control DSST (cDSST) task combination blocks

For the mDSST task, a white fixation-cross appeared for 1 s to target attention, followed by the target digit–symbol combination for 1.2 s. After another white fixation-cross presented for 0.2 s, a block of four digit–symbol combinations was shown for a maximum of 3.6 s. If the target was present among the four combinations, participants were instructed to click as fast as possible with their right, and when not present, with their left index finger (1:1 ratio matching:non-matching). For the cDSST task the target digit–symbol combination was always the same, 2⊥ , and the block of four digit–symbol combinations consisted of the letters H or V (1:1 ratio) instead of the target symbol. When an H (‘Højre’ is Danish for right) was presented, participants were instructed to click as fast as possible with their right, and when a V (‘Venstre’ is Danish for left) was presented with their left index finger. A schematic illustration of cDSST and mDSST tasks is shown in ESM Fig. 2.

positron emission tomographies (PET) imaging

The tracer doses were administered automatically, via a venous cannula in the right arm of participants, using a water injector (Scansys, Denmark) for a period of 60

seconds. Activity decayed for 10 minutes (5 half-lives) before each new tomography session. To ensure that the participants were fully engaged in the cognitive task during acquisition of the PET data, a new cognitive session was initiated (excluding instructions) before the tracer injection. Radioactivity in radial arterial blood samples was recorded with an automatic counter (Allogg, Sweden) with a frequency of once per second during the imaging time of 3 minutes. All scans were performed between 9:00 and 12:00 hours to account for diurnal variation in cerebral blood flow. The participants were imaged in the supine position with their heads fixed in place. Head position was checked prior to each scan using laser marking.

Each participant underwent high-resolution MRI (GE Signa Excite HDx 3T; three-dimensional (3D) fast spoiled grass sequence with IR preparation: echo time=3.0 ms, Inversion time=450 ms, flip angle=20°, slices = 120, slice thickness = 1.5 mm, field of view (FOV) = 240 mm, matrix = 2562) and PET (3D mode: slices = 47, slice thickness = 3.1 mm, FOV = 256 mm, matrix = 1282) imaging. PET images were reconstructed using filtered back-projection with a 0.5 cycles-1 ramp filter, followed by application of a 7-mm Gaussian filter. The regional cerebral blood flow (rCBF) quantification used a 500-MBq bolus injection of $H_2{}^{15}O$ delivered intravenously. The CBF data were acquired in 21 frames (12 × 5 seconds, 6 × 10 seconds, 3 × 20 seconds) over 3 minutes and modelled using single tissue compartment with added vascular space, according to established procedures. Parameter estimation was performed in each participants' native PET space. A transformation between native PET and MRI spaces was calculated for each participant using a six-parameter rigid-body transformation with a mutual information cost function. MRI volumes were finally mapped to a common reference space (MNI 1 mm) with linear and non-linear registrations in BioImage Suite (www.bioimagesuite.com). In addition, MRI images

were skull-stripped and segmented into grey/white/cerebral spinal fluid (CSF) space using FSL BET and FAST (fsl.fmrib.ox.ac.uk). Once in common space, averaged PET maps were computed for different parameters in Matlab (MathWorks, Natick, MA) using custom scripts. While alternative techniques, such as blood oxygenation level-dependent contrast and fMRI, have better spatial and temporal resolution compared to PET $H_2^{15}O$ (as well as no radiation exposure), PET offers the advantages of being quantifiable, less confounded by movement, and allows for serial studies, making this methodology subtler to the scope of the study.

ESM Results

To explore the impact of the perception of hypoglycaemia during the glycaemic clamps (responding yes or no to, 'do you feel hypo') on the primary endpoint during the state of hypoglycaemia, we investigated the differences in mDSST score and mDSST response time between subjects feeling/not feeling hypoglycaemia under hypoglycaemic and euglycaemic clamps. In these relatively small subgroups (n=11, yes (awareness); n=15, no (lack of awareness)) the DSST scores and response times (Mean [±SD]) in participants feeling hypoglycaemia (mDSST score, 29.8 [2.2]; mDSST response time, 41.0 [10.3] s) were slightly lower compared with participants not feeling hypoglycaemia (mDSST score, 30.6 [0.7]; mDSST response time, 41.9 [8.1] s). In addition, in the subgroup feeling hypoglycaemia, the differences in the mDSST score and mDSST response time (between hypoglycaemic and euglycaemic clamps), were similar (mDSST score, -1.0 [1.7]; mDSST response time, 4.0 [7.0] s), compared with the differences between glycaemic clamps in those not feeling hypoglycaemia (mDSST score -0.3 [0.7]; mDSST response time, 1.9 [3.8] s).

ESM Table 1. Inclusion and exclusion criteria.

**Inclusion criteria:**	
Eligible participants had to fulfil all the criteria below.	
1	Informed consent obtained before any trial-related activities. Trial-related activities are any procedures that are carried out as part of the trial, including activities to determine suitability for the trial.
2	Male or female right-handed participants aged 18–64 years (both inclusive).
3	Type 1 diabetes mellitus (as diagnosed clinically) ≥ 12 months.
4	Treated with multiple daily insulin injections or continuous subcutaneous insulin infusion (CSII) ≥ 12 months.
5	Body mass index (BMI) 18.0–28.0 kg/m^2 (both inclusive).
6	Glycosylated haemoglobin (HbA_{1C}) ≤ 9.0 % by local laboratory analysis.
7	Fasting C-peptide ≤ 0.2 nmol/L.
**Exclusion criteria:**	
Eligible participants were not allowed to meet any of the exclusion criteria below	
1	Previous participation in this trial. Participation is defined as screened.
2	Receipt of any investigational medicinal product within 3 month prior to screening in this trial.
3	Participants that have been exposed to ionising radiation or isotopes as part of diagnostic or experimental investigations within the past 5 years.
4	Haemoglobin < 8.0 mmol/L (male) or < 6.4 mmol/L (female), total leukocyte count < 3.0 × 10^9/L, thrombocytes <100 × 10^9/L, plasma creatinine levels ≥ 126 µmol/L (male) or ≥ 111 µmol/L (female), bilirubin > 3 × the upper limit of normal (ULN), alanine aminotransferase (ALAT) and alkaline phosphatase (ALP) > 2 × ULN.
5	Cardiac problems defined as decompensated heart failure (New York Heart Association [NYHA] class III and IV) at any time and/or angina pectoris within the last 12 months and/or acute myocardial infarction at any time.
6	Supine blood pressure at screening (after resting for 5 minutes) outside the range 90−140 mmHg for systolic or 50−90 mmHg for diastolic. This exclusion criterion also pertains to participants taking antihypertensives.
7	Clinically significant abnormal ECG at screening, as judged by the investigator.
8	Visual impairment (i.e. cataract, colour blindness etc.) or auditory impairment.
9	Known abnormalities of the central nervous system (CNS) or any endocrinological (with the exception of diabetes mellitus and euthyroid struma), haematological, neurological, psychiatric diseases or other major disorders that in the opinion of the investigator precludes compliance with the protocol, evaluation of the results or represent an unacceptable risk for the participant's safety.
10	Proliferative retinopathy (funduscopy/fundus photography performed within 12 weeks before the screening visit is acceptable) and/or severe neuropathy.
11	Contraindication to PET or magnetic resonance imaging (MRI) scanning (e.g. suffer from claustrophobia, some metallic implants etc.)
12	Structural brain abnormalities as evident from MRI scanning.
13	Participant who has donated any blood or plasma in the past month or more than 500 mL within 3 months prior to screening.
14	Surgery or trauma with significant blood loss (more than 500 mL) within the last 3 months prior to screening.
15	Current treatment with systemic (oral, intravenously (iv). or inhaled) corticosteroids, monoamine oxidase (MAO) inhibitors, prostaglandin blockers, systemic non-selective beta-blockers, growth hormone, and other drugs, which may interfere with glucose metabolism.
16	Not able or willing to refrain from any use of herbal products and non-routine vitamins within 1 week and routine vitamins within 48 hours prior to start of the experimental visit.
17	Significant history of alcoholism or drug/chemical abuse as per investigator's judgement or a positive result in the urine drug/alcohol screen at the screening visit.
18	Current tobacco user (any smoking or use of nicotinic products within 3 months prior to screening).
19	Severe hypoglycaemic event during the past 6 months or hospitalisation for diabetic ketoacidosis during the previous 6 months.
20	Hypoglycaemic unawareness.
21	Participant with mental incapacity or language barriers precluding adequate understanding or co-operation or who, in the opinion of the investigator or their general practitioner, should not participate in the trial.
22	Female of childbearing potential (menopause: at least 12 months without menstrual cycle) who is pregnant, breast-feeding or intend to become pregnant or are not using adequate contraceptive methods (adequate contraceptive measures include sterilisation, hormonal intrauterine devices, oral contraceptives, spiral, transdermal depot plaster and depot injection).
23	Any chronic disorder or severe disease which, in the opinion of the investigator might jeopardise participant's safety or compliance with the protocol.
**Experimental visit exclusion criteria**	
1	Consumption of alcohol within 24 hours prior to Day 1 of an experimental visit, or positive result of alcohol breath test.
2	Consumption of coffee, tea, chocolate or beverages such as cola and energy drinks containing methylxanthine (caffeine, theophylline or theobromine) within 12 hours prior to Day 1 of an experimental visit.
3	Physical exercise within 24 hours prior to Day 1 of an experimental visit, as judged by the investigator to interfere with trial results.
4	Presence of any medical condition that may confound the results of the trial or pose an unacceptable risk to the participant, as judged by the investigator.
5	Any use of systemic (oral, i.v. or inhaled) corticosteroids, MAO inhibitors, prostaglandin blockers, non-selective beta-blockers, growth hormone and other drugs, which may interfere with glucose metabolism.
6	Any use of herbal products and non-routine vitamins within 1 week prior to Day 1 of an experimental visit and routine vitamins within 48 hours prior to Day 1 of an experimental visit.
7	Hypoglycaemia (PG ≤ 3.9 mmol/L) within 48 hours prior to Day 1 of an experimental visit.
8	Injection of IDeg after 21:00 the evening before the experimental visit Day 1 and/or injection of long or intermediate-acting insulin products after 10:00 at the experimental visit Day 1 and/or injection or bolus infusion of insulin after 19:00 at the experimental visit Day 1. (Note: Participants using continuous subcutaneous insulin infusion (CSII) may arrive at the trial site with their basal rate running).

ESM Table 2. Individual mDSST scores, response times and baseline characteristics for participants with outlier values for mDSST score and/or response time.

Participant ID	mDSST score		mDSST response time		Age (years)	BMI (kg/m^2)	Diabetes duration (years)
	Hypoglycaemia	Euglycaemia	Hypoglycaemia	Euglycaemia			
101037	**28.0**	31.3	49.5	42.3	19.0	25.2	9.7
101032	**24.0**	29.0	**65.5**	42.9	24.0	25.0	13.2
101025	29.3	30.7	**62.1**	53.2	64.6	27.0	46.4

Outlier values from Figure 2a/b are highlighted in bold. The outliers on Figure 2a are 101032 and 101037. The outliers on Figure 2b are 101032 and 101025. ID, identification number; BMI, body mass index; mDSST, modified digit symbol substitution test

ESM Table 3. PASAT results in completers.

	Hypoglycaemia	Euglycaemia
**Number of participants**	26	26
**Number of correct responses, mean (±SD)**		
3-second test	45.9 (9.1)	45.9 (10.3)
2-second test	36.1 (10.1)	35.2 (10.4)

Completers: Participants in the full analysis set with available endpoints at both experimental visits.
Score range: 1–60
PASAT, paced auditory serial addition test; SD, standard deviation

ESM Table 4. Estimated differences in rCBF (given in %) between hypoglycaemic and euglycaemic conditions for cDSST and mDSST.

	cDSST		mDSST	
**Region of interest**	**ETD [95% CI]**	**p-value**	**ETD [95% CI]**	**p-value**
Anterior cingulate gyrus/cortex	0.13 [–2.88; 3.13]	0.9302	–0.02 [–2.63; 2.58]	0.9852
Dorsolateral prefrontal cortex	3.49 [1.84; 5.14]	0.0002	2.89 [0.66; 5.11]	0.0132
Globus pallidus	–4.05 [–9.72; 1.61]	0.1526	–0.65 [–7.02; 5.71]	0.8338
Hippocampus	–2.37 [–4.37; –0.36]	0.0225	–2.59 [–5.17; –0.02]	0.0488
Inferior frontal gyrus	2.94 [1.71; 4.17]	<0.0001	3.10 [1.51; 4.68]	0.0005
Insula temporal lobe	–1.01 [–3.18; 1.16]	0.3451	0.66 [–1.61; 2.93]	0.5565
Medial temporal lobe	–2.08 [–3.27; –0.89]	0.0015	–2.33 [–3.66; –1.01]	0.0013
Middle frontal gyrus	3.26 [1.64; 4.88]	0.0004	2.64 [0.31; 4.98]	0.0281
Orbitofrontal cortex	1.65 [0.34; 2.95]	0.0159	0.85 [–0.78; 2.48]	0.2939
Parahippocampal gyrus	–2.52 [–4.07; –0.96]	0.0028	–2.08 [–4.50; 0.35]	0.0897
Posterior cingulate gyrus	1.46 [–1.76; 4.68]	0.3595	1.28 [–1.70; 4.26]	0.3834
Posterior supramarginal gyrus	0.89 [–1.34; 3.13]	0.4187	1.29 [–1.24; 3.83]	0.3024
Precuneus temporo-parietal	1.12 [–2.02; 4.27]	0.4685	1.22 [–1.48; 3.92]	0.3613
Primary visual cortex	0.34 [–1.87; 2.56]	0.7512	–0.25 [–2.50; 1.99]	0.8175
Striatum	–4.54 [–6.48; –2.60]	0.0001	–0.80 [–2.83; 1.23]	0.4230
Superior frontal gyrus	2.25 [0.40; 4.11]	0.0194	1.22 [–1.07; 3.51]	0.2821
Superior parietal lobe	1.69 [0.38; 3.00]	0.0135	1.42 [0.01; 2.82]	0.0479
Thalamus	8.30 [4.24; 12.37]	0.0003	9.86 [5.90; 13.82]	< 0.0001
Ventromedial prefrontal cortex	2.10 [0.46; 3.75]	0.0145	0.98 [–0.63; 2.59]	0.2218

ETD in rCBF by region of interest normalised to cerebral cortex. Completers: Participants in the full analysis set with available endpoints at both experimental visits. cDSST, control digit symbol substitution test; CI, confidence interval; ETD, estimated treatment difference; mDSST, modified digit symbol substitution test; rCBF, regional cerebral blood flow

ESM Fig. 1. Trial design.

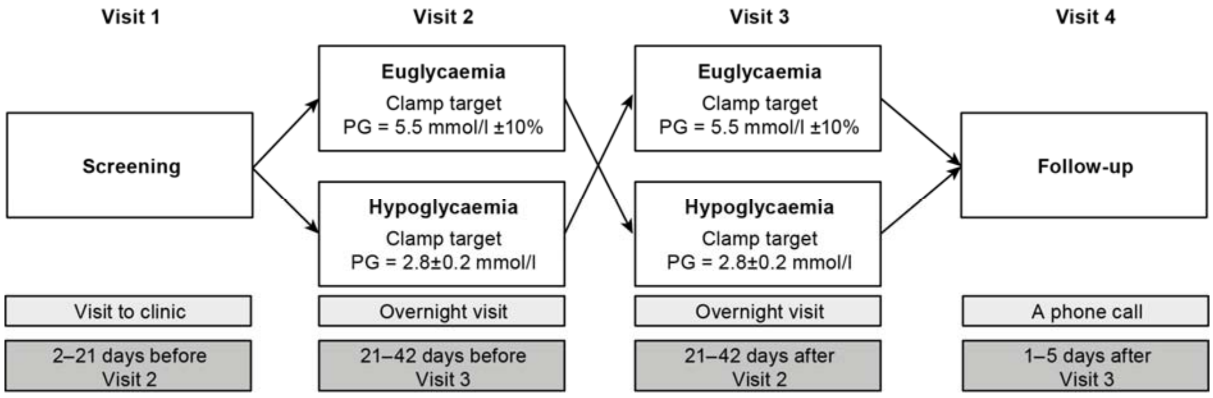

ESM Fig. 2. Schematic illustration of cDSST a. and mDSST b. tasks.

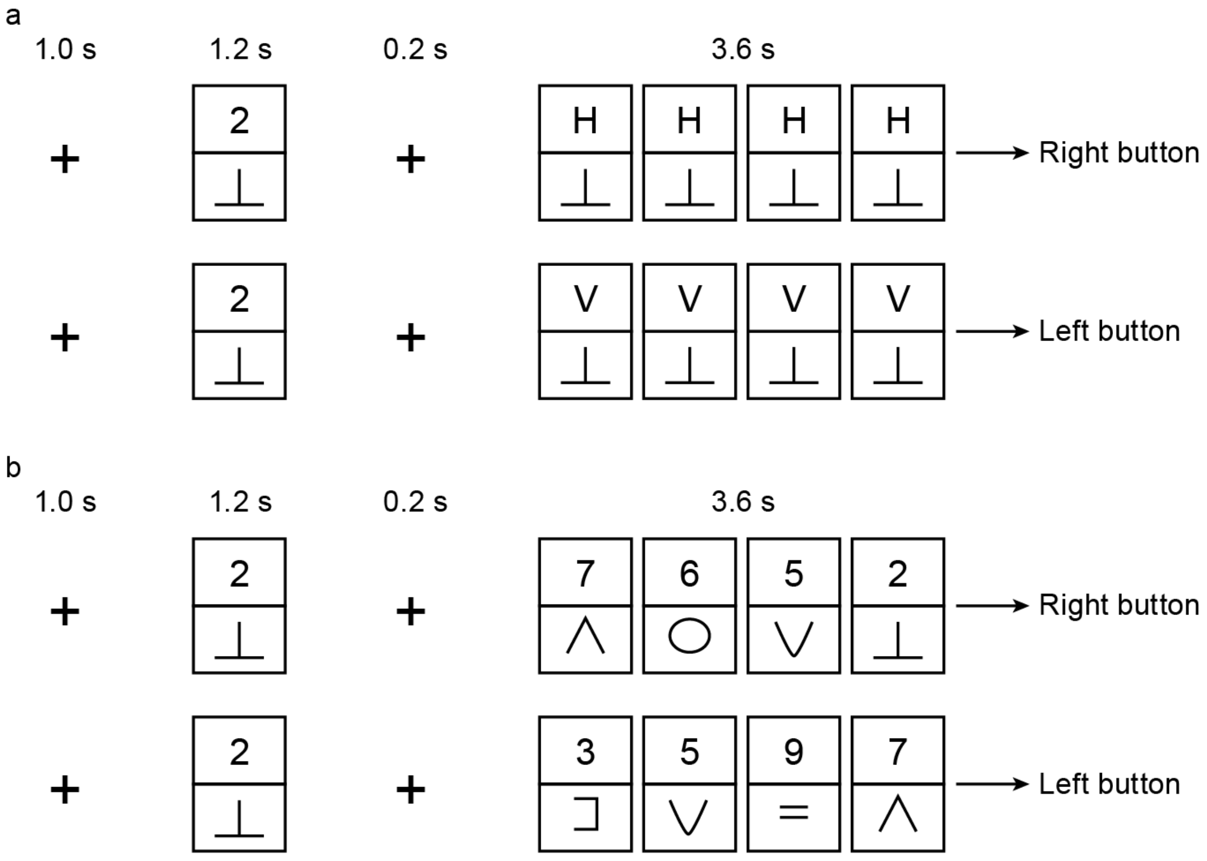

In the cDSST a, participants were to click with their right hand if they were presented with the letter H (‘Højre’ is Danish for right) with a symbol, or click with their left hand if they were presented the letter V (‘Venstre’ is Danish for left) with a symbol. During the mDSST b, participants were first shown a white fixation cross on a black screen for 1 second, after which they were shown a target digit-symbol combination for 1.2 s. Next, a white fixation cross was shown on a black background for 200 ms, after which a block of four digit-symbol combinations was shown for a maximum of 3.6 s. If the target digit-symbol combination was present among the four other combinations, participants had to click with their right hand as fast as possible, if the target was not present, participants had to click as fast as possible with their left hand.

ESM Fig. 3. Participant disposition

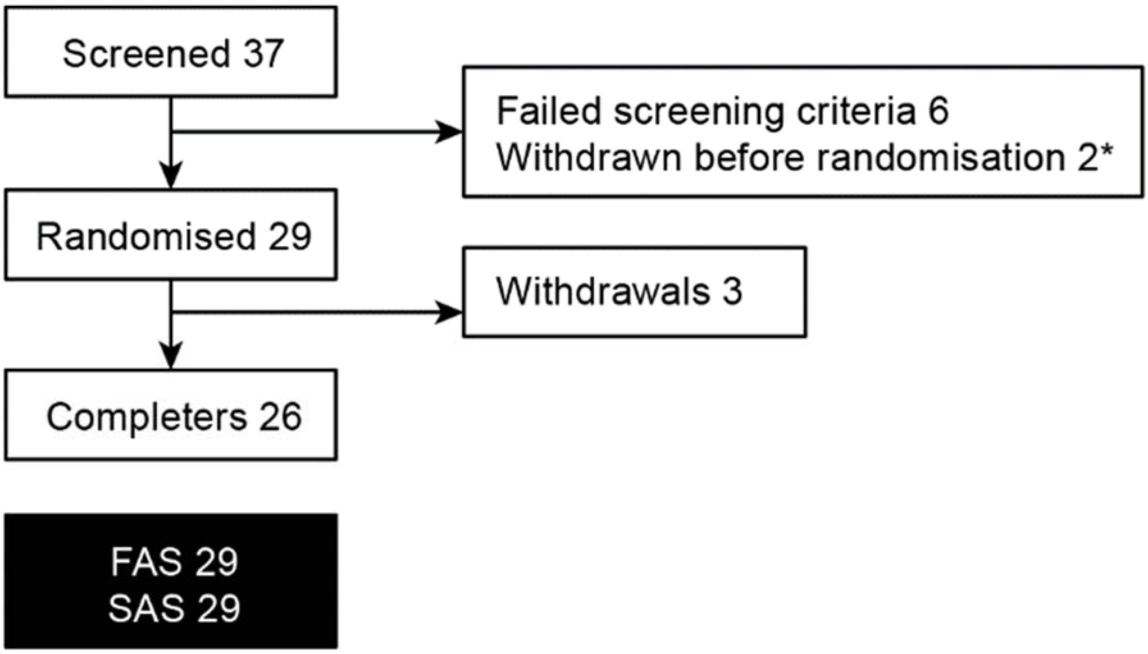

* Two participants met experimental visit exclusion criteria and were not randomised. FAS, full analysis set; SAS, safety analysis set

Reasons for withdrawals: one not possible to place venous catheter, one randomised in error, one met experimental visit exclusion criteria.

ESM Figure 4. Individual plasma glucose profiles during the clamp procedure.

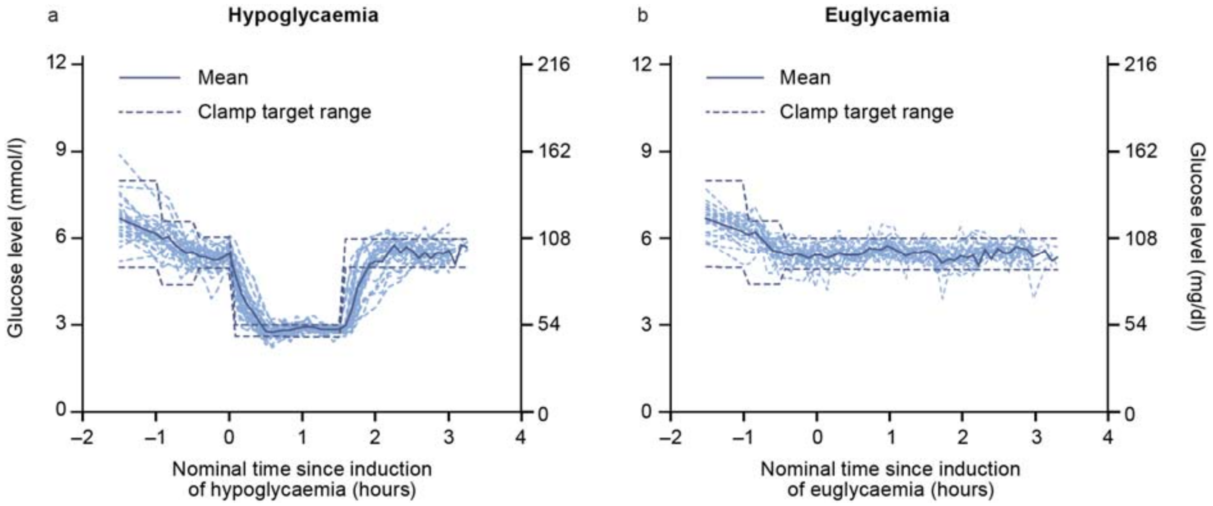

Based on completers. Solid blue line represents the mean glucose concentration. The dashed line represents the reference line.
